# Protein Cargo of Salivary Small Extracellular Vesicles as Potential Functional Signature of Oral Squamous Cell Carcinoma

**DOI:** 10.3390/ijms222011160

**Published:** 2021-10-16

**Authors:** Simona Fontana, Rodolfo Mauceri, Maria Eugenia Novara, Riccardo Alessandro, Giuseppina Campisi

**Affiliations:** 1 Department of Biomedicine, Neurosciences and Advanced Diagnostics, University of Palermo, 90133 Palermo, Italy; novaraeugenia@gmail.com (M.E.N.); riccardo.alessandro@unipa.it (R.A.); 2 Department of Surgical, Oncological and Oral Sciences, University of Palermo, 90127 Palermo, Italy; rodolfo.mauceri@unipa.it (R.M.); giuseppina.campisi@unipa.it (G.C.); 3 Department of Biomedical and Dental Sciences, Morphological and Functional Images, University of Messina, 98124 Messina, Italy; 4 Department of Dental Surgery, Faculty of Dental Surgery, University of Malta, 2090 Msida, Malta; 5 Institute for Biomedical Research and Innovation (IRIB), National Research Council (CNR), 90146 Palermo, Italy

**Keywords:** saliva small extracellular vesicles, liquid biopsy, oral squamous cell carcinoma, protein profiling

## Abstract

The early diagnosis of oral squamous cell carcinoma (OSCC) is still an investigative challenge. Saliva has been proposed as an ideal diagnostic medium for biomarker detection by mean of liquid biopsy technique. The aim of this pilot study was to apply proteomic and bioinformatic strategies to determine the potential use of saliva small extracellular vesicles (S/SEVs) as a potential tumor biomarker source. Among the twenty-three enrolled patients, 5 were free from diseases (OSCC_FREE), 6 were with OSCC without lymph node metastasis (OSCC_NLNM), and 12 were with OSCC and lymph node metastasis (OSCC_LNM). The S/SEVs from patients of each group were pooled and properly characterized before performing their quantitative proteome comparison based on the SWATH_MS (Sequential Window Acquisition of all Theoretical Mass Spectra) method. The analysis resulted in quantitative information for 365 proteins differentially characterizing the S/SEVs of analyzed clinical conditions. Bioinformatic analysis of the proteomic data highlighted that each S/SEV group was associated with a specific cluster of enriched functional network terms. Our results highlighted that protein cargo of salivary small extracellular vesicles defines a functional signature, thus having potential value as novel predict biomarkers for OSCC.

## 1. Introduction

Oral squamous cell carcinoma (OSCC) is one of the most prevalent histotypes of cancer worldwide and is a challenge to public health. Despite the introduction of new diagnostic tools and treatment modalities for the management of OSCC, its prognosis still remains very poor, with a 5-year mortality rate of approximately 60% [1]. Although the accessibility of the oral cavity can render the clinical examination easy, OSCC is usually diagnosed in advanced stages due to diagnostic delay, which obviously decreases the chances of survival [2,3].

To date in current clinical practice, OSCC diagnosis is usually preceded by oral visual examination, including inspection and palpation, by general physicians or dentists. In cases of suspicious neoplastic lesions, the clinical examination is integrated by incisional biopsy followed by histological investigation; however, no specific and reliable molecular markers are yet available [2,4,5]. Thus, more recent research has been focusing on the identification of non-invasive or minimally invasive markers for OSCC screening and longitudinal monitoring of the patients’ response to treatment. In this context, liquid biopsy is a promising method for early diagnosis and real-time monitoring based on the analysis of circulating tumor cells (CTCs), circulating tumor DNAs (ctDNAs), circulating cell-free microRNAs (cfmiRNAs), extracellular vesicles (EVs), and other cancer-derived products isolated by the blood or other biofluids (e.g., saliva, urine, ascites, pleural effusion, etc.) [5,6,7,8]. Liquid biopsy allows one to obtain a real-time picture at different time points, giving information about tumor and tumor burden as well as early evidence of drug resistance and tumor recurrence [4,9], supporting the development of more highly personalized diagnosis and therapies [7,10]. In recent years, several studies have been focused on describing the use of EV-based liquid biopsy as a source of biomarkers for several kinds of cancer [11,12,13,14].

EVs are heterogeneous membranous structures secreted by all living cells, including cancer cells, in the surrounding microenvironment, as well as in proximal and systemic body fluids.

Historically, EVs, based on their biogenesis, were classified in exosomes (of endocytic origin) and microvesicles (directly shed by the plasmatic membrane); however, since it is not always easy to establish the presence of specific markers of subcellular origin, the International Society for Extracellular Vesicles (ISEV) suggests indicating EV subtypes with reference to physical characteristics of EVs, such as the size. Thus, now it is more appropriate to refer to “small EVs” (SEVs, <200 nm) and “medium/large EVs” (M/LEVs) [15].

From a functional point of view, SEVs are described as cell-free messengers playing a crucial role in cell–cell communication, strongly depending on the nature of the transported active biomolecules (proteins, mRNAs, miRNAs, and lipids). A significant body of literature has demonstrated that the SEVs released by tumor cells have an active role in promoting tumor growth and progression [16,17,18] and carry tumor-specific RNAs and proteins that are considered attractive targets for diagnostic application [19,20,21]. Moreover, for their high stability in the circulation and body fluids, SEVs are considered one of the more promising elements characterizing the liquid biopsy. Among the biological fluids, saliva is proposed as an ideal diagnostic medium for biomarker detection. The main advantages of using saliva are its non-invasiveness, ease of collection, and cost-effectiveness, as well as the possibility of detecting low-abundance biomarkers often untraceable in blood or serum samples, which have a more complex molecular composition. In the last 15 years, several studies have widely demonstrated that saliva mirrors the conditions of the oral cavity (as its proximal fluid) but also of the whole body, thus supporting the application of salivary diagnostics for systemic and oral diseases [22,23,24]. Among the components of saliva, SEVs are considered as a specific and stable source of biomarkers, since by reducing the complexity of the whole saliva, they can provide more accurate and clinically relevant information for disease detection and diagnoses [25].

In the last decades, proteomics technologies have represented promising tools for disease-associated biomarker detection, offering the possibility of analyzing the global protein profile of a sample (tumor tissues, body fluids, vesicles). The comparative analysis of protein profiles identified in “normal” and “disease” samples and the following bioinformatic analysis allow one to define a panel of aberrantly expressed proteins that can increase the accuracy of current diagnostic methods.

In this study, we applied proteomic and bioinformatic strategies to determine the potential use of saliva small extracellular vesicles (S/SEVs) derived from OSCC as a potential tumor biomarker source. The proteome profiles of S/SEVs from subjects without OSCC (OSCC_FREE) and from OSCC patients without and with lymph node metastasis (OSCC_NLNM and OSCC_LNM, respectively) were compared using the quantitative proteomic SWATH-MS (Sequential Window Acquisition of all Theoretical Mass Spectra) method. For the first time, this study reveals that the S/SEVs have a specific protein signature differentiating not only healthy controls from OSCC patients but also NLNM patients from LNM ones, showing their potential use as non-invasive liquid biopsies for improving the diagnostic routines and the clinical outcomes of OSCC patients.

## 2. Results

### 2.1. Enrolled Subjects and Sample Collections

**Figure 1:**
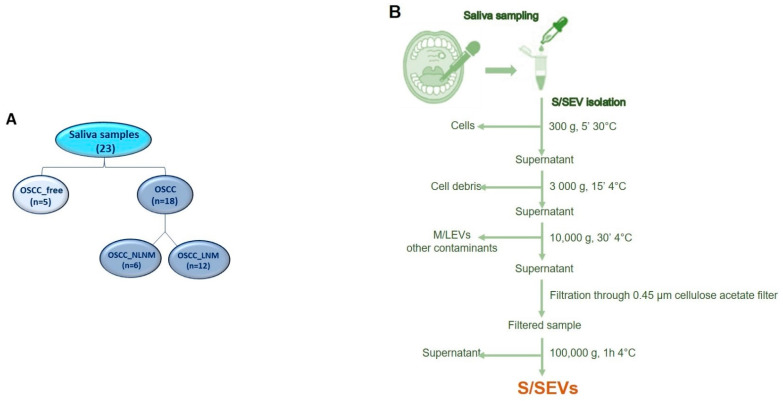
(**A**) Saliva samples used in the study. (**B**) Flowchart of S/SEV purification protocol.

Among the 23 subjects enrolled in this study, 5 were without OSCC (OSCC_FREE group) and 18 were patients with OSCC, of which 6 were without lymph node metastases (NLMN) and 12 with lymph node metastases (LMN) (Figure 1A). Demographic and clinical/anamnestic data of each group are summarized in Table 1. For all groups, the mean age was over 60 years; the OSCC_FREE group was closer to being gender-balanced (# males = 3, 60%; # females = 2, 40%), while a female prevalence was observed in the OSCC_NLNM group (# females = 5, 83.3%) and a male prevalence was observed in the OSCC_LNM group (# males = 8; 66.7%). In the OSCC_FREE group, only one subject was a current or former smoker (20%), while the smokers numbered two (33.3%) and seven (58.3%), respectively, in the OSCC_NLNM and OSCC_NLNM groups. Finally, most of the enrolled subjects were non-drinkers: 100% (5/5) in the OSCC_ FREE group and 83.3% in the OSCC_NLNM and OSCC_LNM groups (respectively, 5/6 and 10/12).

Concerning OSCC sites, in the OSCC_NLNM group, the anterior 2/3 of the tongue was the most commonly affected site (*n* = 5, 83.3%), and the other OSCC affected the gum (*n* = 1, 16.7%). Regarding the OSCC_LNM group, the retromolar area was the most commonly affected site (*n* = 4; 33.3%), followed by the anterior 2/3 of tongue (*n* = 3; 25%), the gum (*n* = 2, 16.7%), the buccal mucosa (*n* = 2; 16.7%), and the floor of the mouth (*n* = 1; 8.3%) (Table 2).

### 2.2. S/SEV Isolation and Protein Cargo Characterization

**Figure 2:**
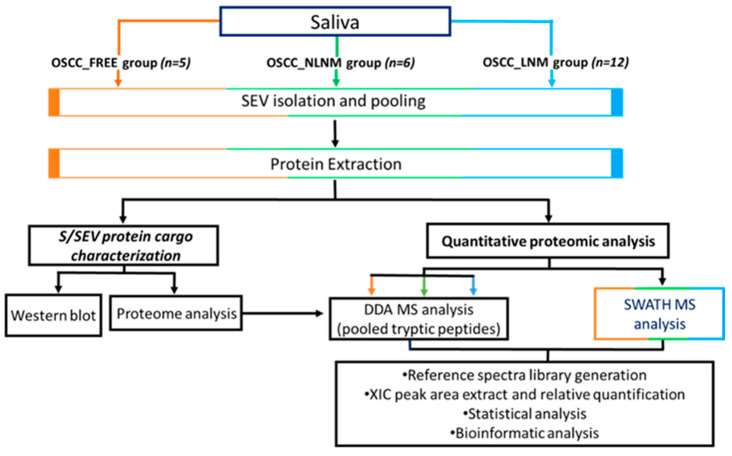
Workflow of the study.

**Figure 3:**
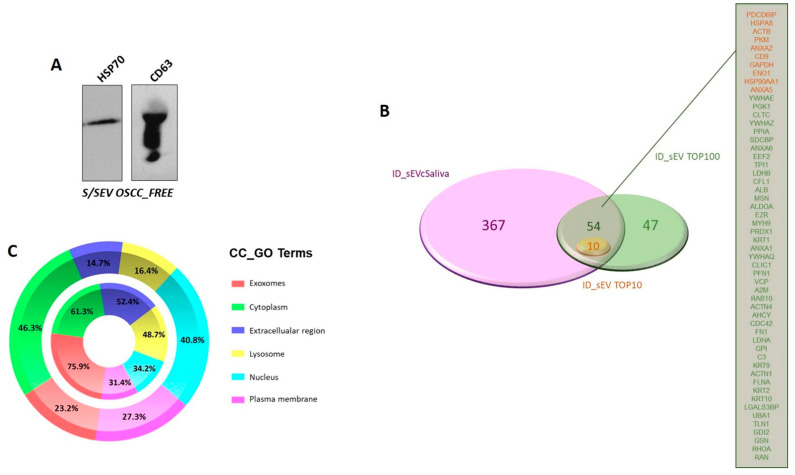
(**A**) Western blot revealing the presence of EV markers in representative in S/SEV OSCC_FREE pooled samples. (**B**) Venn diagram created using the stand-alone enrichment analysis tool FunRich (http://www.funrich.org, accessed on 22 September 2021) showing that among the proteins identified in S/SEV, there were all the TOP10 and more than 50% of the TOP100 EV proteins. (**C**) Percentage distribution of exosome/nanovesicle proteins reported in Vesiclepedia dataset (outer chart) and of the S/SEV proteins (inner chart) within the “Cellular Component” (CC) GO term. The top 6 represented CC_GO terms are reported. The analysis was performed using FunRich.

As reported in the flowchart in Figure 1B, the small EVs were isolated from saliva by performing differential centrifugation and filtration of saliva samples collected from 5 OSCC_FREE subjects and 18 patients with OSCC (6 NLNM and 12 LNM). The EV pellets belonging to the same group were then pooled and used for the analyses summarized in Figure 2. The protein cargo of the isolated SEVs was characterized by evaluating the presence of specific markers. In order to validate the protocol used for S/SEV isolation, we confirmed the presence of the EV markers HSC70 and CD63 in pooled S/SEV OSCC_FREE samples (Figure 3A). Moreover, the obtained reference protein library formed by 421 proteins identified by ProteinPilot 4.5 at a 1% critical against the Homo sapiens UniProt fasta database (Supplementary Table S1—Protein Library and SWATH-MS Data, Sheet “Protein Library” and Table 3) was compared to the Vesiclepedia database by using FunRich software, in order to verify how many TOP10 and TOP100 EV proteins were present within our S/SEV OSCC protein dataset. The Venn diagram in Figure 3B showed that isolated S/SEV contained all the TOP10 and more the 50% of the TOP100 EV proteins. Finally, the analysis performed by FunRich within the GO category “Cellular Component” (GO_CC) showed a good overlapping between the S/SEV protein dataset and the Vesiclepedia dataset referring to exosomes and nanovesicles (Figure 3C). Indeed, we found that the first six most represented terms are the same in the two analyzed datasets, even if there are differences in the percentage of proteins included in each group, probably due the major numeric complexity of the Vesiclepedia dataset.

### 2.3. Protein Profile Characterization of S/SEVs

**Figure 4:**
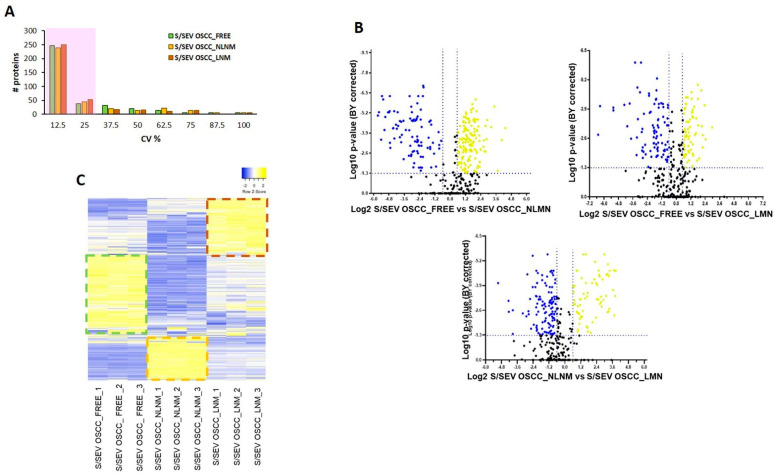
(**A**) Histogram shows the distribution of coefficients of variation (CV) among technical replicates of S/SEV OSCC_FREE, S/SEV OSCC_NLNM, and S/SEV OSCC_LNM. About 80% of the proteins have CV ≤ 25% (shadow area). (**B**) Volcano plot of the log2 fold change (x-axis) versus the -log10 BH corrected *p*-value (y-axis) of the 365 quantified proteins. The dashed lines correspond to 1.5-fold up and down (vertical lines), and a BY corrected *p*-value of 0.05 (horizontal line). In the plots, the yellow dots represent the proteins significantly up-represented and the blue ones the proteins significantly down-represented in the comparison indicated in the x-axis. (**C**) Heat map representing color-coded expression levels of proteins quantified in the three replicates of each group of pooled S/SEVs: yellow indicates high expression values and blue indicates low expression values. Details of regulated genes are provided in Supplementary Table S2.

The obtained spectral reference library was then used for developing the SWATH-MS strategy, and 7852 targeted peptides (filtered using an FDR threshold of ≤5% over nine runs) allowed obtaining of a detection rate of 75.3% (47314 of 62816), resulting in quantitative information for 365 proteins (Supplementary Table S1, sheet “SWATH-MS Data”). We found that among the technical replicates of each group, the percentage of proteins whose quantitation showed a coefficient of variation (CV) ≤25% in the quantitative data was around 80% (Table 3 and Figure 4A).

In our analysis, we considered as differentially modulated proteins those showing a fold change (FC) > ±1.5 (>1.5 or <0.067) in relative abundance and a corrected BY *p*-value ≤ 0.05, indicated as yellow dots (up-represented) and blue (down-represented) in the volcano plots in Figure 4B. In total, as summarized in Table 4, the significantly differentially modulated proteins were 235 in the comparison of S/SEV OSCC_FREE vs. S/SEV OSCC_NLNM (144 up-represented proteins and 91 down-represented), 157 in the comparison of S/SEV OSCC_FREE vs. S/SEV OSCC_LNM (68 up-represented proteins and 89 down-represented), and 189 in the comparison of S/SEV OSCC_ NLNM vs. S/SEV OSCC_LNM (70 up-represented proteins and 119 down-represented). The high number of regulated proteins we found is due not only to the small sample size, but also to the small fold change cutoff that we set for getting a wide overview of differences characterizing each of the analyzed S/SEV groups. This choice served the purpose of highlighting, rather than single proteins, an S/SEV protein profile to which was assigned the value of biomarker for OSCC. For these reasons, in this study we will not present analysis of single proteins, even if highly regulated, since this speculation should require a validation step on single S/SEV preparation. We have retained more useful and valid, according to the kind of used samples, to perform an analysis aimed at extrapolating a protein signature of OSCC S/SEVs. Further analyses will eventually be needed to propose specific proteins which can have a direct role in clinical practice, but this is not the aim of this study.

Details of the performed quantitative analysis are reported in Supplementary Table S2, in sheets “SEV OSCC_FREE vs. SEV OSCC_NLNM”, “SEV OSCC_FREE vs. SEV OSCC_LNM”, and “SEV OSCC_ NLNM vs. SEV OSCC_LNM”, respectively.

The modulation of the all-quantified protein, shown in the heat map in Figure 4C, highlighted that each S/SEV pool is specifically distinguished from the others by the group of proteins that are up-represented, corresponding to yellow bars framed by the dotted line. In light of this observation, among the significantly modulated proteins reported in the volcano plot in Figure 4C (and listed in the Supplementary Table S2), we extrapolated those that in each group (S/SEV OSCC_FREE, S/SEV OSCC_NLNM, and S/SEV OSCC_LNM) were significantly up-represented in comparison to the other two, showing a fold change (FC) ≥ 1.5 with BY *p*-value ≤ 0.05 (Table 5, Table 6 and Table 7).

**Figure 5:**
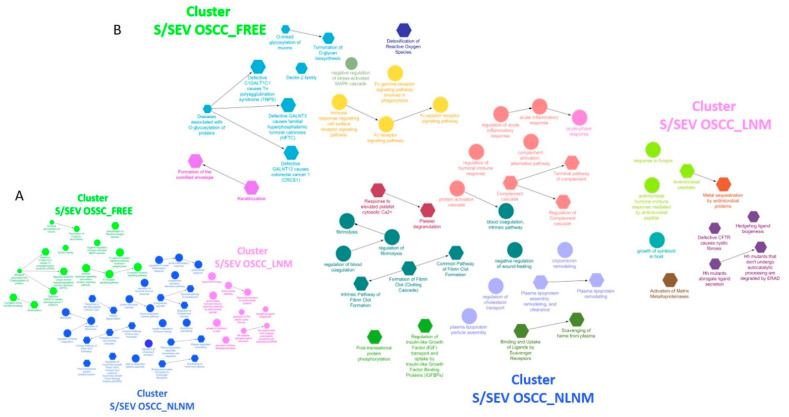
(**A**) ClueGo analysis of the proteins up-represented in each of the three S/SEV subtypes highlighting different clusters of enriched functional network terms (Adj *p*-value < 0.05); (**B**) within each identified cluster, the terms/nodes with the same color form a GO functional groups (see for details Figure 6). Within the clusters, each node represents a term of “biological process” (circle) or a Reactome pathway (hexagon), and the arrows represent direct relations between the nodes. Nodes are specifically related to a cluster when at least the 75% of the proteins of the node belong to that cluster.

**Figure 6:**
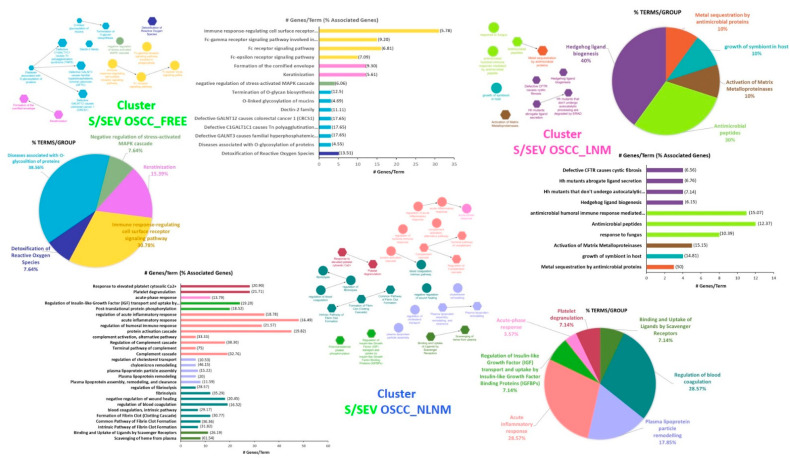
Graphical representation of data obtained by ClueGo analysis. For each S/SEV OSCC cluster, the number of genes/term and the number of terms/functional GO are reported.

The analysis of these up-represented proteins performed using ClueGo allowed us to highlight three different clusters of enriched functional network terms (Adj *p*-value < 0.05) for each of the three S/SEV subtypes (Figure 5A and Supplementary Table S3, sheet “ClueGO Results”), indicated as CLUSTER S/SEV OSCC_FREE, CLUSTER S/SEV OSCC_NLNM, and CLUSTER S/SEV OSCC_ LNM. Within the clusters, each node represents a term of “biological process” (circle) or a Reactome pathway (hexagon), and the arrows represent direct relations between the nodes. Nodes are specifically related to a cluster when at least 75% of the proteins of the node belong to that cluster (Table S3, sheet “ClueGO Results”). In Figure 5B, nodes/terms with the same color form a GO functional group, as specified in Figure 6 and in the Supplementary Table S3 (sheets “CLUSTER OSCC_FREE, “CLUSTER OSCC_NLNM”, and “CLUSTER OSCC_ LNM”). Interestingly, we found that these GO groups were unique for each cluster and defined a specific functional signature of S/SEV OSCC_FREE, S/SEV OSCC_NLNM, and S/SEV OSCC_LNM (Figure 6). Since it is known that the protein cargo of EVs often reflects that of the originating cells, the functional signature characterizing the three clusters can probably mirror the biological status and activities of the oral mucosa cells in the three analyzed clinical conditions. In particular, the ClueGo analysis highlighted five GO groups specifically associated with CLUSTER S/SEV OSCC_FREE, five with CLUSTER S/SEV OSCC_LNM, and seven with CLUSTER S/SEV OSCC_NLNM. Among the five GO groups identified in the CLUSTER S/SEV OSCC_FREE (Figure 6 and Supplementary Table S3), it was interesting to find the group “Detoxification of Reactive Oxygen Species“ (associated to ERO1A, GSTP1, PRDX1, PRDX6, TXN—see Table 5 for the FC in S/SEV OSCC_FREE), the group “Diseases associated with O-glycosilation of proteins” (associated to MUC 5, MUC7, and MUC16—see Table 5 for the FC in S/SEV OSCC_FREE), and the group “Keratinization” (associated to DSG3, KRT1, KRT10, KRT9, PRSS8, SPRR3—see Table 5 for the FC in S/SEV OSCC_FREE), all activities that can protect oral mucosa against cancer development [26,27,28,29,30,31]. The last GO Group associated to the CLUSTER S/SEV OSCC_FREE was that of “Immune-response-regulating cell surface receptor signaling pathway” (associated to several immunoglobulin heavy and light chains, RAP1A, CEACAM1, EZR, MUC16, MUC5B, MUC7, PIGR, PRDX1, RAP1A—see Table 5 for the FC in S/SEV OSCC_FREE), indicating that S/SEV OSCC_FREE are enriched in proteins involved in the modulation of immune response.

Within the CLUSTER S/SEV OSCC NLNM (Figure 6 and Supplementary Table S3), we found several GO groups reflecting well-known conditions associated with OSCC. Indeed, our analysis highlighted that this cluster was characterized by the presence of proteins involved in “acute inflammatory response” (the proteins associated to this GO group were several components of the complement system, some immunoglobulin heavy chains, A2M, AMBP, APCSCFH, CLU, F2, FGA, FGB, FGG, HPX, KLKB1, KNG1, ORM1, PROS1, SERPINs, and VTN—see Table 6 for the FC in S/SEV OSCC_NLNM), a condition characterizing the microenvironment and often modulated by the complement system [32,33]. Of note, the CLUSTER S/SEV OSCC_NLNM was also specifically associated with the GO groups of “regulation of blood coagulation” and “platelet degranulation” (associated to A1BG, A2M, AHSG, ALB, APCS, some apolipoproteins, CLU, ECM1, F2, FGA, FGB, FGG, FN1, HRG, ITIH4, KLKB1, KNG1, ORM1, PLG, PROS1, several SERPINs, TF, VTN—see Table 6 for the FC in S/SEV OSCC_NLNM). Finally, within the CLUSTER S/SEV OSCC_NLNM we found the GO group “plasma lipoprotein particle remodeling” (associated to A2M, ALB, APOA1, APOA2, APOB, APOC1, APOC3, APOE, ALB, APOA4—see Table 6 for the FC in S/SEV OSCC_NLNM).

Among the five GO groups specifically characterizing the CLUSTER S/SEV OSCC_LNM, three were related to activities against pathogens (Figure 6 and Supplementary Table S3): “metal sequestration by antimicrobial proteins” (associated with LCN2, LTF, S100A9—see Table 7 for the FC in S/SEV OSCC_LNM); “growth of symbiont in host” (associated with ELANE, MPO, PGLYRP1—see Table 6 for the FC in S/SEV OSCC_LNM); and “antimicrobial peptides” (associated with ELANE, GAPDH, LCN2, LTF, MPO, PGLYRP1, PI3, PRTN3, RNASE3, S100A12, S100A9—see Table 7 for the FC in S/SEV OSCC_LNM).

## 3. Discussion

An early and accurate diagnosis of OSCC often provides the best chance of survival and favorable outcomes as compared to diagnoses in advanced stages. To date, the visual inspection of the oral cavity followed by an incisional biopsy is still considered the gold standard diagnostic method for OSCC [2]. However, these approaches require the presence of lesions and visible alterations of oral mucosa, often not allowing the early capture of the latent or still asymptomatic malignant lesions. Thus, the availability of molecular biomarkers in the biological fluid becomes indispensable. In this context, blood and saliva EVs (B/EVs and S/EVs respectively) represent a valid source for detection of OSCC biomarkers [34,35,36]. However, even though, due to the emerging exosome technologies, interesting data on the diagnostic and prognostic values of miRNA and protein profiles of EVs has been available [37], many efforts for a deep molecular characterization of EVs are still needed, and further studies have to be performed to allow clinical applications of this knowledge.

In this study, in order to provide new insights leading to the development of valid diagnostic and prognostic tools for OSCC, we performed a proteome quantitative SWATH-MS analysis of S/EVs isolated from healthy subjects and patients with NLNM and LNM OSCC.

Unlike the shot-gun proteomic methods used to investigate S/EV proteomes [34], the targeted SWATH-MS strategy employed in this study is a specific variant of data-independent acquisition (DIA) methods emerging as a technology that combines deep proteome coverage capabilities with quantitative consistency and accuracy, making it a valid strategy for biomarker discovery [38,39].

Results showed that the S/SEV OSCC_FREE, S/SEV OSCC_NLNM, and S/SEV OSCC_LNM were characterized by the enrichment of specific proteins belonging to GO groups which defined a unique functional signature of each S/SEV cluster. Since it is known that the protein cargo of EVs often reflects that of the originating cells, the functional signature characterizing the three clusters can probably mirror the biological status and activities of oral mucosa cells in the three analyzed clinical conditions. As reported in the “Results” section, among the GO groups identified in the CLUSTER S/SEV OSCC_FREE (Figure 6 and Supplementary Table S3), we found the group “detoxification of Reactive Oxygen Species“ (associated to ERO1A, GSTP1, PRDX1, PRDX6, TXN—see Table 5 for the FC in S/SEV OSCC_FREE), the group “diseases associated with O-glycosilation of proteins” (associated to MUC 5, MUC7, and MUC16—see Table 5 for the FC in S/SEV OSCC_FREE), and the group “keratinization” (associated to DSG3, KRT1, KRT10, KRT9, PRSS8, SPRR3—see Table 5 for the FC in S/SEV OSCC_FREE), all activities that can protect oral mucosa against cancer development. Indeed, since it is well known that oxidative stress and consequent ROS production are involved in the pathogenesis of oral cancer [30], the higher presence in S/SEV_FREE of proteins eliciting an anti-oxidative response can mirror the condition of the originating cells, therefore indicating their ability to protect oral mucosa from the pro-tumoral solicitations. Similarly, the higher presence in S/SEV_FREE of MUC 5, MUC7, and MUC16 may indicate a condition in which the oral mucosa of OSCC_FREE subjects is more protected from bacterial infections that are strictly related to oral carcinogenesis [29]. The mucins are highly O-glycosylated proteins forming the mucus gel layers on several organs with a tissue specificity, thus maintaining a continuous defensive barrier protection against all aggressive external forces [26]. In the oral cavity, the mucosal pellicle is mostly composed by the salivary mucins MUC5B, MUC7 (having antifungal, antibacterial, and antiviral functions), and by the secretory IgA (SIgA), which constitutes the main specific immune defense mechanism playing an important role in the homeostasis of the oral microbiota [28]. Due to this composition, the mucosal pellicle works as a protective layer, ensuring lubrication of the oral epithelia and also protection against excessive bacterial colonization [29]. Moreover, it is also known that beside their proper defensive action, mucins mediate the SIgA binding to the mucosal surface, thus influencing the immune activity of the mucosal pellicle [27]. The higher presence of mucins in S/SEV_FREE can indicate a better predisposition to prevent oral dysbiosis that emerging evidence suggests to be involved in oral cancer development [29]. In addition, the presence in the CLUSTER S/SEV OSCC_FREE of the GO group “keratinization” may prompt a condition of well-being of the oral mucosa of OSCC_FREE subjects. Indeed, it is known that in the oral cavity, the keratinocytes, through a network of desmosomes and keratins, form a strong anatomical barrier that protects from both mechanical and chemical stress, as well as from microbial infections [31]. It was interesting to find within this GO group, the Small Proline Rich Protein 3 (SPRR3) recently proposed as a novel diagnostic and prognostic tumor marker of OSCC, since the survival analysis showed that its under-expression was associated to a poor prognosis, and that the decrease of SPRR3 expression corresponded to the increased the tumor malignancy [40].

Within CLUSTER S/SEV OSCC_NLNM, it was interesting to specifically find the GO groups of “regulation of blood coagulation” and “platelet degranulation” (associated to A1BG, A2M, AHSG, ALB, APCS, some apolipoproteins, CLU, ECM1, F2, FGA, FGB, FGG, FN1, HRG, ITIH4, KLKB1, KNG1, ORM1, PLG, PROS1, several SERPINs, TF, VTN) and the GO group “plasma lipoprotein particle remodeling” (associated to A2M, ALB, APOA1, APOA2, APOB, APOC1, APOC3, APOE, ALB, APOA4). Hypercoagulability is a recurrent condition of several types of cancer, causing the venous thromboembolism (VTE) that is a common complication in patients with cancer [41]. Thus, it was stimulating to find that CLUSTER S/SEV OSCC_NLNM was characterized by the presence of proteins associated with the coagulation process, which distinguished this cluster not only from that of S/SEV OSCC_FREE, but also from that of S/SEV OSCC_LNM.

The role of lipid carriers in cancers is widely discussed, and emerging evidence highlights that the functionality and the impact of the apolipoproteins on the tumor microenvironment depend on the specific tissue context [42]. Interestingly, it was reported that stress-induced recruitment of lipoproteins and EVs represents a new mechanism of cancer cell adaptation, and that microenvironment changes induced by tumor cells can promote the formation of EV/lipoprotein complexes affecting the following entry and cargo transfer into recipient cells [43].

Finally, the CLUSTER S/SEV OSCC_LNM was specifically associated to GO groups related to activities against pathogen agents, such as “metal sequestration by antimicrobial proteins” (associated to LCN2, LTF, S100A9—see Table 6 for the FC in S/SEV OSCC_LNM); “growth of symbiont in host” (associated to ELANE, MPO, PGLYRP1—see Table 6 for the FC in S/SEV OSCC_LNM); and “antimicrobial peptides” (associated to ELANE, GAPDH, LCN2, LTF, MPO, PGLYRP1, PI3, PRTN3, RNASE3, S100A12, S100A9—see Table 6 for the FC in S/SEV OSCC_LNM). Proteins of these groups, such as lactoferrin (LFT,), lipocalin-2 (LCN2), S100A9 (forming with S100A8 the heterodimeric complex calprotectin), neutrophil elastase (ELANE), peptidoglycan recognition protein 1 (PGLYRP1), and myeloperoxidase (MPO), are widely described for their antibacterial activity or for their role as inflammatory markers [44,45,46,47,48]. The enrichment of these proteins in the S/SEVs from patients with OSCC_LNM could be indicative of dysbiotic signatures occurring during tumor progression. Evidence accumulated in the last years indicates that alterations of the oral microbiome can have a role in inducing oral cancer progression [49,50,51,52]. Interestingly, some of these proteins (as S100 proteins and LCN2) are described as diagnostic and prognostic markers for several types of tumors, even though their role in oral cancer is controversial [48,53,54].

Taken together, obtained data support the use of S/SEVs as a promising diagnostic marker source for OSCC. Our approach presented here also has limitations, particularly with regard to the small number of patients enrolled and the numerical non-homogeneity of the groups analyzed, so further analyses must be performed using larger data sets. Furthermore, since this proteomic study was performed on S/SEV pools, the validity of the predictive value of their protein cargo in OSCC will also have to be evaluated on single samples.

## 4. Materials and Methods

### 4.1. Subject Enrolment and Saliva Collection

All participants were recruited from the Unit of Oral Medicine at the “Paolo Giaccone” Policlinico University Hospital in Palermo (Italy). The study protocol, which conformed with ethical guidelines of the 1964 Declaration of Helsinki and later amendments or comparable ethical standards, was approved by Institutional Ethics Committee of “Paolo Giaccone” Policlinico University Hospital in Palermo (Approval date: 6 February 2013; approval number 3/2013). All patients signed written informed consent before specimens were collected for the analyses. In total, 18 patients diagnosed with OSCC and 5 subjects OCSS_FREE that were not on any medication and practiced regular oral hygiene were enrolled.

All OSCC patients underwent surgery, including wide tumor excision and neck lymph node dissection and foe. Among them, 6 did not have lymph node metastasis (NLNM) and 12 did (LNM). Finally, three different groups were defined for the following analyses (Figure 1A): the OSCC_FREE group (*n* = 5), OSCC_NLNM group (*n* = 6), and OSCC_LNM group (*n* = 12). All subjects were asked to refrain from eating, drinking, or oral hygiene for at least one hour prior to collection. The volunteers were asked to rinse their mouth with 10 mL water with 0.9% saline to remove food debris and then waited for at least 5 min before collection of about 15 mL of saliva in 50 mL Falcon tubes. Once collected, the saliva samples were immediately kept on dry ice and transported from the hospital to the laboratory for processing. If not immediately processed, the samples were stored at −80 °C until further analyses.

### 4.2. Saliva SEV Isolation

Each saliva sample was diluted 1:1 with phosphate-buffered saline (PBS) prior to proceed to SEV isolation following the experimental workflow shown in Figure 1B. As reported, the saliva samples were centrifuged at 300× *g* for 5 min at 30 °C to eliminate the cells. Then, the supernatant was centrifugated at 3000× *g* for 15 min at 4 °C, and further at 10,000× *g* for 30 min at 4 °C to eliminate cell debris, other contaminants, and M/LEVs as well. Finally, the supernatant was filtered through a 0.45 μm VWR^®^ Vacuum Filtration System (VWR International, West Chester, PA, USA), before ultracentrifugation (Ti70 or Ti45 rotor, Beckman Coulter, Brea, CA, USA) at 100,000× *g* for 70 min at 4 °C to pellet the SVEs, which were finally resuspended in 100 µL PBS.

In order to improve the protein amount and also to minimize the individual-to-individual differences, the SEV pellets isolated from saliva samples (S/SEVs) of the same group were pooled. Thus, subsequent analyzes were carried out on three types of pooled samples: (a) S/SEV OSCC_FREE; (b) S/SEV OSCC_NLNM; and (c) S/SEV OSCC_LNM.

### 4.3. Western Blot

An aliquot of S/SEV OSCC_FREE sample was treated with RIPA lysis buffer with protease inhibitor cocktail [55]. Subsequently, lysates were centrifuged at 12,000 g for 1 h in ice, the supernatant was collected, and the protein concentration was determined by Bradford assay (Pierce, Rockford, IL, USA). Proteins were then separated using 4–12% Novex Bis-Tris SDS-acrylamide gels (Invitrogen, Waltham, Massachusetts, USA) and immunoblotted with the following primary antibodies: CD63 and HSC70 (Santa Cruz Biotechnology, Inc., Santa Cruz, CA, USA). The secondary antibodies were obtained from Santa Cruz Biotechnology (Santa Cruz Biotechnology, Inc., Santa Cruz, CA, USA). Chemiluminescence was detected using Amersham ECL Western Blotting Detection Reagent (Global Life Sciences Solutions, UK Amersham place, Little Chalfont, Buckinghamshire).

### 4.4. Proteomic Analyses: In-Solution Protein Digestion and SWATH-MS Analysis

Pooled S/SEVs (100 µg) were subjected to in-solution digestion using 50% 2,2,2-trifluoroethanol (TFE) in PBS, and obtained peptides were desalted by solid phase extraction using Thermo Scientific Pierce C18 Spin Columns (Thermo Fisher Scientific) [56].

Equal amounts of peptides from each of the three samples were mixed to prepare a pool of tryptic peptides, which was subjected to Data-Dependent Acquisition (DDA) analysis. The resulting list of proteins/peptides was used for construction of the SWATH-MS reference spectral library.

The analysis was performed by a Triple TOF 5600 Plus System equipped with an Eksigent Ekspert nano LC 425 system (AB Sciex, Framingham, USA).

Pooled tryptic peptides (4 ug) were loaded in a C18 reverse-phase trap column (Acclaim PepMap 100 C18 LC Trap Column Thermo Fisher Scientific) at a flow rate of 5 μL/min, using 0.1% *v/v* formic acid (FA) in water from a loading pump. Peptides were then separated on the Acclaim™ PepMap™ RSLC (75 µm × 25 cm nanoViper C18 2 µm 100 Å, Thermo Fisher Scientific) equilibrated at 40 °C with 0.1% FA in water (solvent A) at a flow rate of 300 nL/min) using 0.1% FA in ACN (solvent B), in accord with the following gradient method: linear increase of solvent B from 10 to 40% for 60 min and from 40% to 70% for 15 min, further increase to 95% within 1 min, and maintenance at 95% for 5 min to rinse the analytical column. Finally, decrease of solvent B from 95 to 10% within 1 min, and hold at 10% for remaining 18 min to re-equilibrate the column.

The mass spectrometer operated in MS scan (400 m/z to 1250 m/z; accumulation time 250 ms) in high resolution mode (>30,000) and in MS/MS scan (230 m/z to 1500 m/z; accumulation time 65 ms) in high sensitivity mode (resolution > 15,000) with rolling collision energy. A maximum of 50 precursors per cycle from each MS spectrum, with charge states from 2 to 5, were fragmented if exceeding a threshold of 100 counts per second (cps), with a dynamic exclusion window of 12 s.

The DDA file was submitted to Protein Pilot™ 4.5 software (AB SCIEX, Toronto, Canada); Uniprot was used as the human protein database (downloaded in May 2020, 149,644 protein sequence entries). The database search was performed with the Paragon algorithm by using the following parameters: iodoacetamide cysteine alkylation, digestion by trypsin, and ID focus on biological modifications.

For SWATH-MS analysis, 2 µg of each sample was analyzed in triplicate to avoid random variation by the following SWATH-MS mode: at a cycle time of 2 s, 50 ms TOF/MS survey scan was performed between 400 and 1250Da with 34 × 25 Da precursor isolation window (swath). SWATH MS/MS acquisition was carried out using a 76 ms accumulation time between 230 and 1500 Da. 

### 4.5. Bioinformatic and Statistical Analysis

To evaluate the global protein composition of the S/SEV, the reference protein library obtained by the DDA analysis was compared with the Vesiclepedia database by using the stand-alone enrichment analysis tool FunRich (Functional Enrichment analysis tool; http://www.funrich.org, accessed on 22 September 2021) [57].

Data from SWATH-MS analysis were processed by Peak View v2.2 and Marker View 1.2.1 (AB SCIEX; Framingham, USA). In Peak View, data were analyzed using the following parameters: 10 peptides, 7 transitions per peptide, 90% peptide confidence threshold, 5% false discovery rate threshold (FDR), exclude modified peptides, extracted ion chromatogram (XIC) extraction window of 5 min, 0.05 Da XIC width. The protein list with FDR lower than 5% generated by analyzing SWATH-MS data with PeakView 2.2 was exported to MarkerView for normalization of protein intensity (peak area) using the total area sums algorithm and *t*-test analysis [58]. Proteins were considered to be differentially expressed if the fold change (FC) among the compared groups was >±1.5 (>1.5 or <0.067) with corrected *p*-value ≤ 0.05.

The analyses of coefficients of variation, mean calculation, and Student’s *t*-test were performed by using Microsoft Excel 2016. Mean of the replicates was used to perform the following comparisons: (a) S/SEV OSCC_FREE vs. S/SEV OSCC_NLNM; (b) S/SEV OSCC_FREE vs. S/SEV OSCC_LNM; (c) S/SEV OSCC_NLNM vs. S/SEV OSCC_LNM. GraphPad Prism 9.00 for Windows was used for (i) performing the *p*-value Benjamini–Yekutieli correction (BY *p*-value); (ii) to make a volcano plot scaling in which the FC was transformed using the log2 function, so that the data is centered on zero, while the BY corrected *p*-value was −log10 transformed [57]. The expression-based heat map was obtained by using the Heatmapper freely available web server (http://www.heatmapper.ca, accessed on 22 September 2021), applying the following criteria: (a) clustering method: average linkage; (b) distance measurement method: Kendall’s tau. To identify the biological processes and functional pathways specifically correlated to the protein cargo of S/EV OSCC_FREE, S/EV OSCC_ NLNM, and S/EV OSCC_ LNM, the bioinformatic tool ClueGO v2.5.2 + CluePedia v1.5.2, a Cytoscape v3.8.0 plug-in was used. This analysis allowed us to visualize the non-redundant gene ontology (GO) terms (within the term “biological processes”) and functional pathways (searched in Reactome pathway database) in organized networks reflecting the relations between the biological groups based on the similarity of their linked genes/proteins [59]. In order to make a group comparison and highlight functional differences, the three protein groups of up-regulated proteins were uploaded in ClueGO as separate clusters using the Cytoscape environment [60]. For the enrichment of biological terms and functional groups, we used the two-sided (enrichment/depletion) test based on the hyper-geometric distribution. We set the statistical significance to 0.05 (*p* ≤ 0.05), and we used the Benjamini–Hochberg adjustment to correct the *p*-value for the terms/groups visualized by ClueGO. We used fusion criteria to diminish the redundancy of the terms shared by similar associated proteins. The used parameters were: kappa score threshold set to 0.4; GO tree interval: 3–8; GO Term Fusion.

## 5. Conclusions

In conclusion, our study provides new evidence highlighting a S/SEV-based protein functional signature specifically associated to the absence of OSCC as well as to the LNM or LMN status, thus having a potential application value as novel predictive biomarkers for OSCC. The increase of sample size and the development of a validation phase based on targeted DIA strategies (as selected reaction monitoring), immunoassays, and so on, will be necessary to validate the S/SEV protein signature and the clinical value proposed.

## Figures and Tables

**Table 1:** Demographic and clinical/anamnestic data of the enrolled patients.

Anamnestic Data		OSCC_FREE Group (*n* = 5)	OSCC_NLNM Group (*n* = 6))	OSCC_LNM Group (*n* = 12)
Mean Age		61.4 (±11.2)	68.2 (±7.8)	67.4 (±9.6)
Gender				
	Male	3 (60%)	1 (16.7%)	8 (66.7%)
	Female	2 (40%)	5 (83.3%)	4 (33.3%)
Smoking Habit				
	Non-smokers	4 (80%)	4 (66.7%)	5 (41.7%)
	Smokers	1 (20%)	2 (33.3%)	7 (58.3%)
Alcohol Consumption				
	Non-drinkers	5 (100%)	5 (83.3%)	10 (83.3%)
	Former drinkers	0	1 (16.7%)	2 (16.7%)

**Table 2:** OSCC site in NLNM and LNM group.

Group	Age	Sex	OSCC Site	Grading	TNM	Stage
**OSCC_NLNM**	F	69	Anterior 2/3 of tongue	G2-G3	T1N0M0	I
	F	58	Anterior 2/3 of tongue	G2-G3	T2N0M0	II
	F	70	Anterior 2/3 of tongue	G3	T2N0M0	II
	F	67	Anterior 2/3 of tongue	G3	T1N0M0	I
	F	83	Gum	G2-G3	T2N0M0	II
	M	62	Anterior 2/3 of tongue	G2	T2N0M0	II
**OSCC_LNM**	F	78	Gum	G2	T2N1M0	III
	M	63	Retromolar area	G2-G3	T4aN2bM0	IVA
	M	77	Buccal mucosa	G2	T3N2M0	IVA
	F	74	Anterior 2/3 of tongue	G2	T2N2aM0	IVA
	M	73	Retromolar area	G2-G3	T3N1M0	III
	F	66	Gum	G2-G3	T4aN2aM0	IVA
	M	48	Retromolar area	G2	T3N2cM0	IVA
	F	82	Buccal mucosa	G2-G3	T3N1M0	III
	M	54	Anterior 2/3 of tongue	G3	T2N1M0	III
	M	67	Anterior floor of mouth	G3	T4aN2M1	IVC
	M	66	Anterior 2/3 of tongue	G3	T2N2cM0	IVA
	M	61	Retromolar area	G3	T3N2bM1	IVC

**Table 3:** Summary of proteome analysis.

	S/SEV OSCC_FREE	S/SEV OSCC_NLNM	S/SEV OSCC_LNM
Number of identified proteins(DDA protein library)	421		
Number of proteins quantified in SWATH-MS analysis	365		
Number (and percentage) of quantified protein with CV ≤ 25% among technical replicates	284 (78%)	284 (78%)	303 (83%)

**Table 4:** Summary of proteome quantitative analysis.

	S/SEV OSCC_FREE vs. S/SEV OSCC_NLNM	S/SEV OSCC_FREE vs. S/SEV OSCC_LNM	S/SEV OSCC_NLNM vs. S/SEV OSCC_LNM
Number of modulated proteins	235	157	189
Number of up-regulated proteins	144	68	70
Number of down-regulated proteins	91	89	119

**Table 5:** Proteins specifically up-represented in S/SEV OSCC_FREE.

PROTEINS UP-REPRESENTED in S/SEV OSCC_FREE vs. S/SEV OSCC_NLNM and S/SEV OSCC_LNM				
	S/SEV OSCC_FREE vs. S/SEV OSCC_NLNM		S/SEV OSCC_FREE vs. S/SEV OSCC_LNM	
Gene Name	FC	BY *p*-Value	FC	BY *p*-Value
A2ML1	4.219	4.76 × 10^−6^	4.225	1.85 × 10^−5^
ARHGDIB	20.046	5.94 × 10^−5^	2.205	7.94 × 10^−4^
B4GALT1	2.344	5.92 × 10^−5^	2.381	2.26 × 10^−5^
BPIFB2	4.057	2.66 × 10^−4^	1.774	1.29 × 10^−2^
CD59	2.406	7.49 × 10^−6^	1.713	1.32 × 10^−3^
CEACAM1	1.539	1.01 × 10^−2^	2.306	3.65 × 10^−3^
CSTB	3.717	1.79 × 10^−4^	1.599	2.85 × 10^−3^
DPP4	4.911	4.00 × 10^−4^	2.517	1.85 × 10^−4^
DSG3	3.780	1.40 × 10^−3^	1.576	5.78 × 10^−3^
ERO1A	3.741	1.02 × 10^−4^	4.327	7.30 × 10^−4^
EZR	4.487	1.66 × 10^−2^	2.570	1.21 × 10^−2^
FABP5	2.839	7.34 × 10^−4^	2.038	1.39 × 10^−3^
FCGBP	3.124	3.82 × 10^−6^	1.782	7.47 × 10^−5^
GDI2	4.233	7.87 × 10^−5^	2.101	2.39 × 10^−2^
GLRX	3.345	8.31 × 10^−4^	1.514	1.55 × 10^−2^
GSTP1	3.986	8.28 × 10^−7^	2.429	4.43 × 10^−5^
IGHA1	1.682	3.01 × 10^−4^	1.600	1.35 × 10^−2^
IGHA2	3.181	4.30 × 10^−5^	1.630	9.69 × 10^−4^
IGHV1-2	3.206	1.63 × 10^−4^	1.645	2.01 × 10^−3^
IGHV1-8	3.395	1.81 × 10^−4^	1.700	2.16 × 10^−3^
IGHV3-15	2.833	2.06 × 10^−5^	3.462	4.43 × 10^−5^
IGHV3-23	3.340	1.67 × 10^−6^	3.433	1.06 × 10^−5^
IGHV3-7	2.175	8.91 × 10^−5^	2.790	9.93 × 10^−5^
IGHV3-72	2.703	2.01 × 10^−2^	3.301	2.48 × 10^−2^
IGHV3-9	2.832	3.01 × 10^−4^	2.500	2.67 × 10^−4^
IGHV4-31	3.132	1.40 × 10^−4^	2.467	1.67 × 10^−4^
IGHV5-51	3.084	1.49 × 10^−3^	2.591	4.67 × 10^−3^
IGKC	1.645	1.03 × 10^−2^	1.596	1.77 × 10^−2^
IGKV1-13	16.563	3.57 × 10^−4^	7.833	8.21 × 10^−4^
IGKV2-24	2.669	8.37 × 10^−5^	2.641	2.99 × 10^−4^
IGKV2D-28	3.104	3.95 × 10^−4^	2.124	2.03 × 10^−3^
IGKV3-7	1.867	3.35 × 10^−4^	1.808	4.58 × 10^−4^
IGKV4-1	2.346	5.56 × 10^−6^	1.919	3.84 × 10^−5^
IGLV1-47	2.801	2.63 × 10^−5^	1.718	1.28 × 10^−2^
IL36A	2.991	3.74 × 10^−4^	2.180	3.70 × 10^−4^
KLK1	2.371	3.57 × 10^−4^	1.789	1.84 × 10^−3^
KLK11	2.869	7.32 × 10^−3^	1.883	2.90 × 10^−2^
KRT1	6.860	4.30 × 10^−5^	3.514	6.68 × 10^−4^
KRT10	3.864	2.32 × 10^−5^	3.157	3.29 × 10^−4^
KRT9	9.278	5.09 × 10^−5^	5.088	8.53 × 10^−5^
LEG1	7.471	1.78 × 10^−5^	2.021	4.70 × 10^−4^
MIF	2.618	1.41 × 10^−5^	1.737	3.83 × 10^−5^
MUC16	2.237	1.64 × 10^−3^	3.081	1.51 × 10^−3^
MUC5B	2.996	1.29 × 10^−4^	1.652	1.21 × 10^−3^
MUC7	4.333	9.18 × 10^−5^	1.968	9.74 × 10^−4^
PAM	2.954	4.11 × 10^−3^	1.650	4.89 × 10^−2^
PDCD6IP	3.097	6.34 × 10^−4^	2.913	1.80 × 10^−3^
PFN1	4.645	4.67 × 10^−5^	1.539	4.63 × 10^−3^
PIGR	3.157	5.56 × 10^−6^	1.712	7.35 × 10^−5^
PRDX1	2.745	1.50 × 10^−5^	1.682	1.12 × 10^−4^
PRDX6	2.902	1.52 × 10^−3^	1.995	5.45 × 10^−3^
PROM1	3.190	7.64 × 10^−4^	1.613	8.08 × 10^−3^
RAP1A	2.686	4.76 × 10^−4^	1.848	2.03 × 10^−3^
SERPINB13	4.951	9.62 × 10^−3^	4.588	1.82 × 10^−3^
SERPINB3	3.054	8.31 × 10^−4^	1.618	8.09 × 10^−3^
SERPINB5	3.617	2.09 × 10^−5^	2.574	1.36 × 10^−4^
SPRR3	5.290	1.49 × 10^−3^	2.303	8.08 × 10^−3^
TFF3	3.929	5.57 × 10^−3^	2.641	1.49 × 10^−2^
TXN	4.129	1.76 × 10^−3^	2.003	9.17 × 10^−3^
YWHAZ	3.510	1.75 × 10^−5^	1.592	1.28 × 10^−4^
ZG16B	11.069	2.42 × 10^−6^	1.548	2.01 × 10^−4^

**Table 6:** Proteins specifically up-represented in S/SEV OSCC_NLNM.

PROTEINS UP-REPRESENTED in S/SEV OSCC_NLNM vs. S/SEV OSCC_FREE and S/SEV OSCC_LNM				
	S/SEV OSCC_NLNM vs. S/SEV OSCC_FREE		S/SEV OSCC_NLNM vs. S/SEV OSCC_LNM	
Gene Name	FC	BY *p*-Value	FC	BY *p*-Value
A1BG	2.798	2.10 × 10^−2^	1.592	2.75 × 10^−4^
A2M	6.270	1.74 × 10^−5^	2.210	6.61 × 10^−4^
AHSG	6.747	4.30 × 10^−4^	1.876	3.21 × 10^−3^
ALB	4.256	1.36 × 10^−7^	2.626	2.88 × 10^−6^
AMBP	14.291	4.88 × 10^−4^	2.456	5.62 × 10^−3^
APCS	16.885	4.30 × 10^−5^	5.393	1.17 × 10−4
APOA1	8.000	5.56 × 10^−6^	1.668	1.28 × 10^−4^
APOA2	7.601	2.06 × 10^−5^	1.646	7.63 × 10^−4^
APOA4	12.525	6.10 × 10^−3^	3.344	3.29 × 10^−2^
APOB	4.968	1.66 × 10^−5^	4.149	9.25 × 10^−5^
APOC1	33.965	1.13 × 10^−5^	4.655	5.97 × 10^−5^
APOC3	5.447	2.16 × 10^−2^	5.776	6.82 × 10^−4^
APOE	5.997	2.06 × 10^−4^	2.907	6.02 × 10^−4^
APOH	5.406	8.31 × 10^−4^	7.510	7.17 × 10^−4^
C1R	8.320	3.84 × 10^−3^	9.948	2.81 × 10^−3^
C1S	21.978	5.91 × 10^−5^	6.646	4.85 × 10^−4^
C3	8.758	1.43 × 10^−5^	2.998	9.25 × 10^−5^
C4A	7.062	1.10 × 10^−2^	13.261	4.62 × 10^−3^
C4B	13.800	1.63 × 10^−4^	14.008	4.44 × 10^−4^
C4BPA	47.980	5.59 × 10^−06^	13.112	2.01 × 10^−5^
C4BPB	22.217	6.19 × 10^−5^	6.869	1.60 × 10^−3^
C5	8.230	3.04 × 10^−3^	1.962	3.29 × 10^−2^
C6	35.474	7.87 × 10^−5^	3.882	3.77 × 10^−2^
C7	7.835	2.23 × 10^−4^	2.299	5.19 × 10^−3^
C8A	20.753	2.79 × 10^−4^	2.112	3.70 × 10^−3^
C9	7.681	2.04 × 10^−3^	2.666	3.39 × 10^−4^
CD5L	6.049	4.71 × 10^−4^	1.975	1.52 × 10^−3^
CFB	4.768	8.31 × 10^−4^	6.263	1.15 × 10^−3^
CFH	4.943	4.27 × 10^−3^	3.210	4.59 × 10^−4^
CLU	1.810	7.34 × 10^−4^	2.291	6.55 × 10^−4^
ECM1	1.876	1.08 × 10^−4^	1.685	1.20 × 10^−3^
F2	20.900	4.76 × 10^−6^	9.344	3.13 × 10^−5^
FGA	50.711	9.10 × 10^−6^	14.394	2.08 × 10^−5^
FGB	38.017	8.28 × 10^−7^	7.917	2.88 × 10^−6^
FGG	29.326	5.65 × 10^−6^	10.431	2.08 × 10^−5^
FN1	14.280	2.32 × 10^−5^	5.879	2.08 × 10^−5^
GC	5.916	3.35 × 10^−4^	2.567	2.69 × 10^−3^
GLUL	2.553	5.29 × 10^−4^	5.317	6.49 × 10^−4^
HABP2	18.099	6.53 × 10^−5^	12.473	2.17 × 10^−4^
HBA1	4.942	3.79 × 10^−6^	1.524	1.50 × 10^−4^
HBB	4.413	5.07 × 10^−7^	1.850	2.08 × 10^−5^
HBD	2.964	1.33 × 10^−3^	2.189	6.93 × 10^−3^
HP	9.067	5.07 × 10^−7^	2.988	2.88 × 10^−6^
HPR	5.949	4.76 × 10^−4^	1.875	2.92 × 10^−3^
HPX	4.703	5.01 × 10^−5^	2.413	5.15 × 10^−5^
HRG	17.313	5.56 × 10^−6^	6.654	9.27 × 10^−4^
IGHG1	5.954	1.47 × 10^−6^	1.899	2.08 × 10^−5^
IGHG2	9.230	8.15 × 10^−5^	3.302	3.05 × 10^−4^
IGHG3	15.347	2.31 × 10^−4^	7.226	5.95 × 10^−5^
ITIH1	6.718	2.00 × 10^−5^	2.244	6.62 × 10^−4^
ITIH2	18.967	3.54 × 10^−5^	5.154	9.25 × 10^−5^
ITIH4	8.286	2.42 × 10^−6^	4.882	7.04 × 10^−6^
KLK13	1.811	1.25 × 10^−3^	1.578	1.33 × 10^−4^
KLKB1	26.394	5.30 × 10^−4^	9.351	3.13 × 10^−3^
KNG1	6.160	1.43 × 10^−5^	5.107	6.15 × 10^−3^
LPA	26.553	5.07 × 10^−7^	5.994	3.38 × 10^−5^
ORM1	27.811	5.65 × 10^−6^	9.577	9.54 × 10^−6^
PLG	26.842	4.76 × 10^−6^	10.061	3.91 × 10^−5^
PROS1	11.100	7.87 × 10^−5^	1.581	1.22 × 10^−3^
SERPINA3	15.356	2.09 × 10^−5^	2.224	1.33 × 10^−3^
SERPINA4	4.895	2.10 × 10^−2^	8.646	4.44 × 10^−4^
SERPINC1	12.622	2.00 × 10^−5^	8.810	3.38 × 10^−5^
SERPIND1	3.400	1.14 × 10^−3^	2.006	3.67 × 10^−3^
SERPINF2	25.834	1.14 × 10^−3^	10.267	2.06 × 10^−3^
TF	5.764	2.53 × 10^−3^	1.991	2.57 × 10^−2^
VTN	40.677	5.07 × 10^−7^	6.165	4.22 × 10^−5^

**Table 7:** Proteins specifically up-represented in S/SEV OSCC_LNM.

PROTEINS UP-REPRESENTED in S/SEV OSCC_NLNM vs. S/SEV OSCC_FREE and S/SEV OSCC_LNM				
	S/SEV OSCC_NLNM vs. S/SEV OSCC_FREE		S/SEV OSCC_NLNM vs. S/SEV OSCC_LNM	
Gene Name	FC	BY *p*-Value	FC	BY *p*-Value
ACTN1	1.641	3.62 × 10^−3^	2.970	1.25 × 10^−3^
ACTR3	1.570	6.66 × 10^−4^	3.768	4.85 × 10^−4^
ANXA3	1.951	8.08 × 10^−3^	2.258	4.60 × 10^−3^
AZU1	13.474	2.60 × 10^−3^	2.253	1.34 × 10^−2^
CA6	1.869	4.86 × 10^−3^	3.846	6.77 × 10^−4^
CST5	1.509	6.38 × 10^−3^	2.083	1.01 × 10^−3^
ELANE	14.603	7.16 × 10^−5^	2.648	1.25 × 10^−4^
FCN1	2.756	1.09 × 10^−4^	4.392	4.63 × 10^−4^
FTL	3.868	6.66 × 10^−4^	3.202	4.39 × 10^−2^
GAPDH	2.107	1.33 × 10^−2^	2.718	6.49 × 10^−3^
H2BC21	9.628	2.30 × 10^−3^	2.076	4.03 × 10^−3^
KLK10	1.695	2.27 × 10^−2^	1.944	5.40 × 10^−3^
KLK14	2.870	3.90 × 10^−4^	6.661	4.03 × 10^−3^
LCN2	1.614	1.09 × 10^−4^	2.041	9.99 × 10^−4^
LTA4H	1.553	1.66 × 10^−2^	5.109	1.42 × 10^−2^
LTF	9.363	1.39 × 10^−5^	1.659	5.97 × 10^−5^
MMP9	1.698	2.74 × 10^−2^	2.376	3.90 × 10^−2^
MPO	10.720	1.08 × 10^−6^	2.478	2.88 × 10^−6^
MYL6	2.264	4.11 × 10^−3^	1.654	2.91 × 10^−3^
NUCB2	3.670	2.25 × 10^−3^	19.150	7.94 × 10^−4^
PGLYRP1	1.659	3.29 × 10^−4^	2.814	9.25 × 10^−5^
PI3	19.846	8.17 × 10^−5^	2.694	3.12 × 10^−3^
PRB1	89.610	1.74 × 10^−3^	18.859	3.00 × 10^−3^
PRR27	4.359	1.67 × 10^−4^	5.401	1.50 × 10^−4^
PRTN3	2.127	2.78 × 10^−4^	4.468	2.08 × 10^−5^
PSMA5	3.158	1.67 × 10^−2^	3.295	2.32 × 10^−2^
PSMB2	5.007	4.67 × 10^−2^	1.730	3.89 × 10^−2^
RETN	2.731	7.47 × 10^−5^	1.676	9.25 × 10^−5^
RNASE3	16.775	7.30 × 10^−4^	4.570	4.44 × 10^−4^
S100A12	37.287	1.44 × 10^−4^	4.506	1.74 × 10^−3^
S100A9	3.173	4.43 × 10^−5^	4.128	2.74 × 10^−5^
SCGB3A1	2.108	1.03 × 10^−4^	3.282	6.47 × 10^−5^
SMR3B	3.503	6.35 × 10^−3^	15.324	2.49 × 10^−3^
TIMP1	2.154	7.47 × 10^−5^	5.408	3.40 × 10^−6^
TKT	2.904	1.46 × 10^−2^	7.511	5.17 × 10^−3^
TMEM198	80.329	9.30 × 10^−5^	33.058	9.25 × 10^−5^
VCP	2.052	8.87 × 10^−3^	1.922	8.65 × 10^−3^

## Data Availability

Data is contained within the article or supplementary material.

## Supplementary Materials

Supplementary materials can be found at https://www.mdpi.com/article/10.3390/ijms222011160/s1.
